# Value coding by primate amygdala neurons complies with the continuity axiom of economic choice theory

**DOI:** 10.1152/jn.00574.2025

**Published:** 2026-02-13

**Authors:** Fabian Grabenhorst, Wolfram Schultz, Simone Ferrari-Toniolo

**Affiliations:** 1 Department of Experimental Psychology, University of Oxford, Oxford, UK; 2 Department of Physiology, Development and Neuroscience, University of Cambridge, Cambridge, UK

**Keywords:** Amygdala, choice, subjective value, decision-making, economic axiom

## Abstract

The primate amygdala contributes to decision-making by encoding the subjective value of rewards, but whether these signals align with principles of economic theory remains unclear. Here, we tested compliance of amygdala value-coding with the continuity axiom of Expected Utility Theory (EUT), which posits a trade-off between reward probability and magnitude, in two male macaques. Given three ranked gambles, axiom-compliance was assessed via the monkeys’ behavioral indifference between the intermediate gamble and a probabilistic combination of the other two. Choices reflected probability-magnitude integration to scalar subjective values consistent with the axiom. In a non-choice task, amygdala neurons showed graded responses to probability and magnitude cues that reflected these individual preferences, equalizing at subjective indifference. During choice, amygdala neurons directly integrated these value components and translated them into chosen-value and choice signals. These findings demonstrate that amygdala neurons represent behaviorally relevant, preference-based values in accordance with EUT’s continuity axiom, and contribute to translating these values into economic decisions.

## Introduction

Economic decision-making involves assigning subjective values to choice options and comparing these values between options. Although many studies implicated neurons in specific brain areas in economic valuation and decision processes (1–11), fewer studies examined directly whether neuronal value signals comply with axioms of economic choice theory (12–17). Such axioms define the necessary and sufficient conditions for maximizing reward and thus are fundamental for understanding economic choice. Combining neurophysiology with formal economic approaches enables testing abstract decision models at the level of single neurons, offering mechanistic insights into observed choices.

The primate amygdala, a nuclear complex in the medial temporal lobe, has been implicated both in assigning value to stimuli and in economic decisions. Primate amygdala neurons encode the values of visual stimuli (18–29), reward timing and contingency (19, 30), economic values and choices in both individual (7, 20, 31, 32) and social contexts (33, 34), probability and magnitude of expected rewards (35), internally generated choice plans (36, 37), and well-defined decision computations (32). Human neuroimaging studies further link amygdala activity to economic decision variables (38–41). Despite these findings, formal testing of whether amygdala value signals conform to economic axioms has been lacking. In contrast, neurons in connected brain areas—the orbitofrontal cortex, dopaminergic midbrain and insula—have been shown to code subjective values consistent with economic principles (12–16, 42, 43).

In Expected Utility Theory (EUT), specific axioms govern value-maximizing choice (44). Completeness (axiom I) and transitivity (axiom II) ensure consistent ranking of options, whereas continuity (axiom III) implies the existence of a numerical value function underlying choice. The independence axiom (axiom IV) specifies how reward magnitudes and probabilities combine to yield expected utility. Together, these axioms provide necessary and sufficient conditions for behavior to be described as maximizing subjective economic value. Empirical violations of the independence axiom in humans and animals (45–47) are accounted for by non-expected utility models, such as prospect theory (48), which relax independence while preserving the continuity requirement.

Here we tested whether the responses of amygdala neurons recorded in non-choice and choice situations are consistent with the continuity axiom of EUT (44). We focus on the continuity axiom because it implies the existence of a continuously varying subjective value function and thus, unlike other axioms, directly constrains the neural representation of value itself. Compliance at the neural level would therefore indicate that amygdala responses to reward magnitude and probability cues encode a scalar economic value, a prerequisite for utility-based decision models. In choices between gambles (options varying in outcome probability and magnitude), the continuity axiom formalizes the integration of reward components into a continuous subjective value quantity. According to the axiom, given three subjectively ranked gambles, a decision-maker should be indifferent between the intermediate gamble and a probabilistic combination of the other two. This indifference point provides a precise behavioral measure of subjective value and reflects the assumption that no option is infinitely more desirable than another, enabling quantitative valuation (49). Continuity is a fundamental principle of economic valuation that underpins most modern economic choice theories, including EUT extensions that address its empirical limitations (50–52).

Axiomatic tests define the function of values in choices that serve to maximize reward. If neuronal signals comply with such axioms, they do not just correlate with values but physically implement them in a way that explains how economic choices maximize reward. Although we previously found that amygdala neurons carry distinct signals for reward probability and magnitude (35), it remains unknown whether these signals can be neuronally integrated into formally defined value signals that reflect interchangeability and integration of probability and magnitude into the subjective value of economic choice options according to the continuity axiom. The aim of our study was therefore to assess whether neuronal responses reflected the encoding of values consistent with choice behavior (i.e., behavior-matching values). In other words, we tested whether neurons reflected integration of subjectively weighted magnitude and probability information, as opposed to the objective expected value or other combinations of the reward variables.

Using behavioral and neuronal tests aligned with the continuity axiom, we derived directly comparable subjective value estimates from both behavior and single-neuron responses. Our results demonstrate that amygdala neurons encode subjective value signals that align with reward-maximizing behavior, supporting their role as neural substrates of formal economic valuation.

## Materials and Methods

### Animals and ethical approval

Two adult male rhesus monkeys (Macaca mulatta) weighing 10.5 and 12.3 kg were used in the experiments. The animals had free access to standard laboratory-macaque diet before and after the experiments and, during recording periods, received their main liquid intake in the laboratory. All animal procedures conformed to US National Institutes of Health Guidelines. The work has been regulated, ethically reviewed and supervised by the following UK and University of Cambridge (UCam) institutions and individuals: UK Home Office, implementing the Animals (Scientific Procedures) Act 1986, Amendment Regulations 2012, and represented by the local UK Home Office Inspector; UK Animals in Science Committee; UCam Animal Welfare and Ethical Review Body (AWERB); UK National Centre for Replacement, Refinement and Reduction of Animal Experiments (NC3Rs); UCam Biomedical Service (UBS) Certificate Holder; UCam Welfare Officer; UCam Governance and Strategy Committee; UCam Named Veterinary Surgeon (NVS); UCam Named Animal Care and Welfare Officer (NACWO).

### Task training

Following habituation to the laboratory environment and experimental set-up, we trained the animals over successive steps to drink liquid reward from the spout, place their hands on a touch-sensitive key and hold the touch key for increasingly longer periods to receive reward, to view different visual conditioned stimuli that resulted in reward delivery, to touch and choose between visual stimuli on a touch screen, to choose between visual stimuli based on fixed stimulus-associated reward probability or cued reward magnitude, to choose between visual stimuli under conditions of varying reward probability or magnitude, to choose between stimuli that varied in both reward probability and reward magnitude, to perform the task under head-fixation, to perform the task under gradually increasing visual fixation requirements including saccade choices. These steps were followed by systematic training in the Pavlovian and choice tasks that would be used during the neurophysiological recording sessions. We progressed from task training to recording once the animals were implanted with recording chambers and when their performance had reached an asymptotic level. These training periods, including development of the tasks, lasted approximately 24 and 18 months for animals A and B.

### Non-choice (Pavlovian) task used for neurophysiological recordings

**Figure 1:**
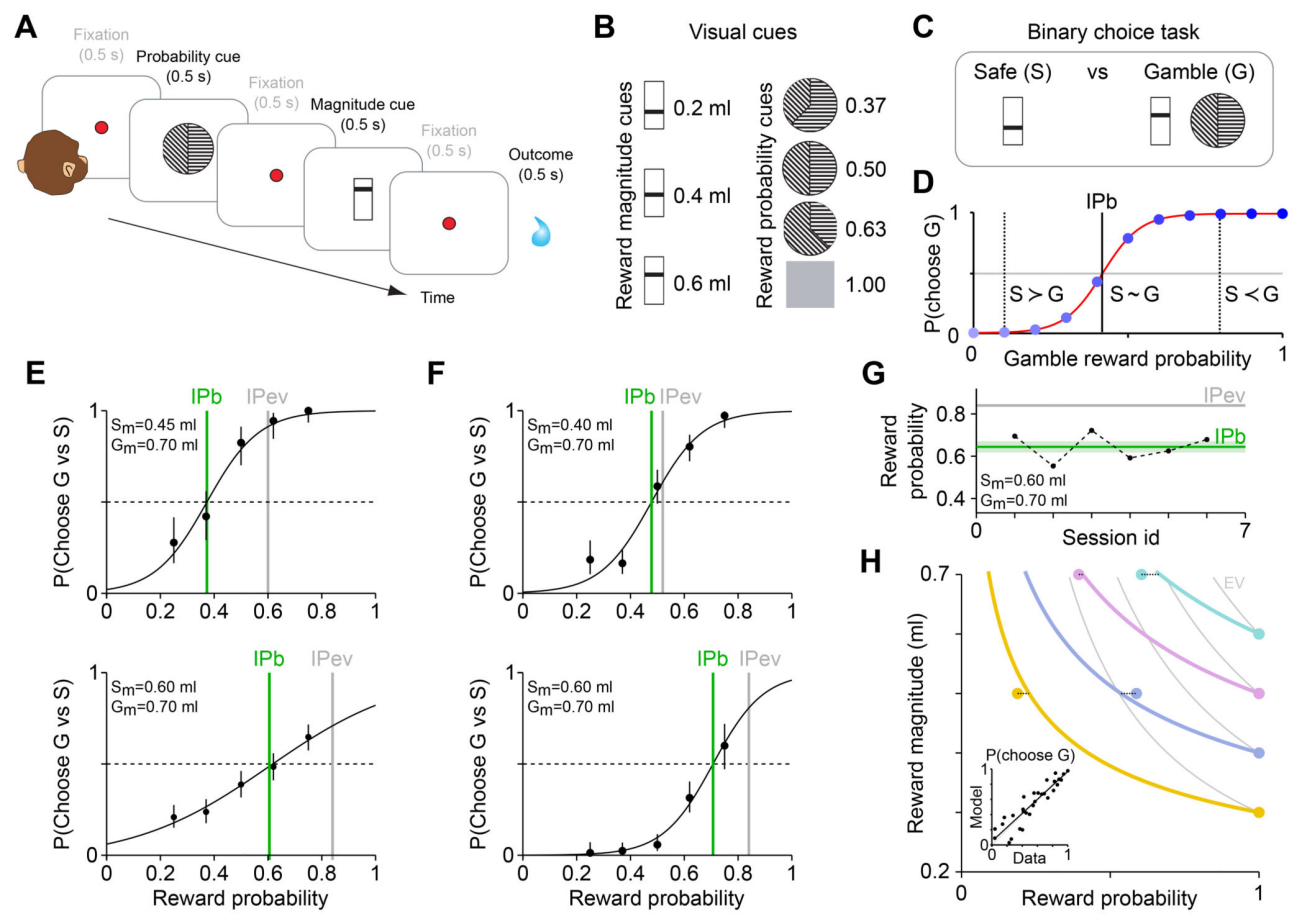
Experimental design and behavioral data. A) Trial structure. Pavlovian task for neural recordings: each trial started with the appearance of a red dot (fixation spot), which the monkey was required to fixate until reward delivery. A reward probability cue, a fixation spot and a reward magnitude cue appeared in successive time intervals. These intervals lasted 500 ms (0.5 s) each. After a further 500 ms fixation period, the reward outcome was delivered, contingent on the indicated reward magnitude and probability cues. B) Visual cues. The vertical position of a horizontal bar represented the reward magnitude (m) information, while a circular stimulus conveyed the probability (p) information. C, D) Indifference point estimation. Through binary choices between a fixed safe option and gambles with variable reward probability (C), we estimated the behavioral indifference point (IPb) as the reward probability for which the monkey was indifferent between the two options (D). We fitted a softmax function (red curve) to the proportion of gamble choices (blue dots) and identified the IPb as the gamble reward probability for which the softmax value was 0.5. This procedure was based on the continuity axiom of EUT, which states that a continuous variation of the reward probability should result in preferences shifting from the safe towards the gamble option, passing through an indifference point. The continuity axiom implies the existence of a continuously varying subjective value function. E-G) Elicited indifference points. Example behavioral indifference points (IPb, green line) elicited though binary choices between a safe option (magnitude Sm) and a gamble option (magnitude Gm) with varying reward probability (Gp). Dots: proportion of trials in which the gamble was chosen; vertical bars: binomial 95% confidence intervals. IPev (grey line): indifference point computed from objective reward values (i.e., probability Gp for which the gamble’s expected value (EV = Gm · Gp) equals the safe magnitude). An IPb lower than the IPev indicated a risk seeking attitude (i.e., gamble preferred to safe option with equal EV). Varying the safe magnitude from 0.45 ml (top) to 0.60 ml (bottom), resulted in a higher IPb, as expected from a monotonically increasing value function. Data from all sessions of monkey A (panel E) and monkey B (panel F); variability of IPb across different behavioral sessions (panel G), for one example test in monkey A. H) Indifference map. Choices between safe and gamble options with different Gm and Sm values resulted in a set of behavioral indifference points. We defined an indifference curve as all points with equal subjective value, as opposed to an expected value (EV) curve connecting points with equal objective mathematical expected value. Estimating an economic value function from behavioral choices (maximum likelihood procedure, see Methods), allowed us to compute a set of indifference curves, which theoretically validated and interpolated the estimated IPb’s. The four colored indifference curves correspond to four fixed safe magnitude levels (0.3, 0.4, 0.5 and 0.6 ml). Inset: significant correlation between measured and economic model-estimated choice probabilities (R^2^ = 0.84, P = 1.3e-12, N = 30). Data from monkey A.

Two monkeys performed in a Pavlovian task with sequentially presented conditioned stimuli indicating the probability (as first cue) and magnitude (as second cue) of predicted liquid rewards under computer control (Fig. 1A-C). As a convention, probability cues were presented first. The monkey sat in a primate chair (Crist Instruments) in front of a horizontally mounted touch screen for stimulus display (EloTouch 1522L 15’; Tyco). Each trial started when the background color on the touch screen changed from black to gray. To initiate the trial, the monkey had to place his hand on an immobile, touch-sensitive key. Presentation of the gray background was followed by presentation of a central ocular fixation spot (1.3° visual angle). The animal was then required to fixate this spot within 4° for 500 ms and maintain fixation until reward delivery. The fixation spot was followed by presentation of a visual conditioned stimulus in the centre of the screen for 500 ms that indicated reward probability, drawn from a set of six stimuli (Fig. 1C). The probability stimuli were monochrome circular ‘sector’ stimuli; each sector stimulus consisted of two sectors distinguished by black-white shading at horizontal and oblique orientation with the amount of horizontal shading indicating the probability of obtaining the cued reward magnitude. Each stimulus predicted forthcoming reward with a specific probability of P = 0.37, P = 0.5, P = 0.63, or P = 0.75. Reward probability of P = 1 was cued by a gray square. This first cue period was followed by a 500-ms inter-stimulus interval which was followed by the second cue period, in which we presented a monochrome bar stimulus with the vertical position of the black horizontal bar against a white background indicating the magnitude of the predicted reward (drawn from a set of three magnitudes: 0.2, 0.4, 0.6 ml). A 500-ms inter-stimulus interval followed the second cue period before reward delivery. Reward delivery was followed by a trial-end period of 1,000 – 2,000 ms which ended with extinction of the gray background. The next trial started after an inter-trial interval of 2,000 – 4,000 ms (drawn from a uniform random distribution). A recording for a given neuron would typically last 90 trials.

Experimental conditions varied pseudo-randomly on a trial-by-trial basis. The specific reward probabilities and magnitudes were chosen based on pre-testing to ensure that the animals maintained high motivation during the task while at the same time providing sufficient variation to study neuronal activities related to probability, magnitude, expected value and risk. Together, the combination of the reward probability and reward magnitude cue on a given trial specified a probability distribution of possible reward magnitudes that could be delivered on that trial. Accurate reward prediction required the monkeys to combine information about the transiently cued reward probability and magnitude internally. A computer-controlled solenoid valve delivered liquid (juice) reward from a spout in front of the monkeys’ mouth. On each completed trial (without fixation breaks), the monkey received one of two outcomes: on ‘rewarded’ trials, we delivered a liquid reward corresponding to the cued reward magnitude in ml whereas on ‘non-rewarded’ trials, a small reward of 0.05 ml was delivered. We used small rewards rather than non-reward as we found that a small reward ensured that the animals maintained high motivation during recordings.

Possible errors in performance included failure to make contact with the touch-sensitive key before the trial, key release before trial completion, failure to fixate the central fixation spot at trial start or fixation break in the period between initial fixation and reward delivery. Errors led to a brief time out (3,000 ms) with a black background followed by trial repetition. We usually interrupted task performance after three consecutive errors. Fixation was continually monitored by the task program during all of these periods and fixation breaks resulted in an error trial. The animals were required to place their hand on a touch-sensitive key to initiate each trial and keep their hand in place on the key until trial completion.

Stimuli and behavior were controlled using custom MATLAB code (The Mathworks) and Psychophysics toolbox (version 3.0.8). The laboratory was interfaced with data acquisition boards (NI 6225; National Instruments) installed on a PC running Microsoft Windows 7.

### Behavioral choice task

We performed a separate choice task using the stimuli from the Pavlovian task to study the monkeys’ preferences for reward probabilities, reward magnitudes and associated expected value and risk levels and to confirm that the monkeys could use the information provided by these stimuli to make meaningful, reward-maximizing choices (preferring higher over lower reward probabilities and higher over lower reward magnitudes). The choice task was performed during the period of neurophysiological recordings in the same monkeysbut typically on separate testing days, on separate testing days, in order to maximize trial numbers for both behavioral choice tests and for each recorded neuron on neurophysiological recording days. In the choice task, reward magnitude was represented the same bar stimuli used in the main task and probability of reward was conveyed the same fractal or sector stimuli, presented adjacent to the bar stimulus. On each trial, the animal made a choice between two gambles, one of which was a ‘safe’ option, or ‘degenerate gamble’ (reward probability of P = 1, trial-by-trial varying reward magnitudes), presented randomly in left-right arrangement on the monitor. The safe option was cued only by a reward-magnitude cue, implying a reward probability of P = 1. For risky gambles, the cued reward magnitude could be obtained with the cued probability and a fixed small reward (0.05 ml) could be obtained with P = 1 – cued probability. Each trial started when the background color on the touch screen changed from black to gray. Trial start was similar to the main task. After 500 ms, the two choice options appeared on the touch monitor in left-right arrangement, followed after 750 ms by presentation of two blue rectangles below the choice options at the margin of the monitor, close to the position of the touch-sensitive key on which the animal rested its hand. The animal was then required to touch one of the targets within 1,500 ms to indicate its choice. Once the animal’s choice was registered, the unchosen option disappeared and after a delay of 500 ms, the chosen object also disappeared and a liquid reward was given to the animal. Reward delivery was followed by a trial-end period of 1,000 ms which ended with extinction of the gray background.

### Choice task used for neurophysiological recordings

The task has previously been described in detail (32). Two monkeys performed in a reward-based choice task with sequentially presented choice options under computer control (Fig. 5A). On each trial, the animal made a choice between two sequentially presented options. Each option consisted of a visual ‘object’ (fractals, abstract images, photographs of natural objects such as flowers) presented in central position on the computer monitor overlaid by a small bar stimulus. Two different objects were associated with specific reward probabilities that varied across the testing session without notification. Different bar heights cued different reward magnitudes chosen randomly on each trial. To maximize reward, the animals were required to learn and track the (uncued) reward probabilities associated with the different objects and combine these probability estimates with the trial-specific cued reward magnitudes for the different objects. Reward probabilities varied in blocks of 15-40 trials (remaining constant within each block) and were pseudorandomly chosen for each object from the following set: 0, 0.15, 0.35, 0.5, 0.65, 0.75, 0.85, 1.0. Reward magnitudes varied randomly on each trial and were chosen from the following set: 0.25 ml, 0.4 ml, 0.65 ml. On each completed trial, the animal received one of two outcomes: on ‘rewarded’ trials, a liquid reward corresponding to the cued reward amount in ml was delivered whereas on ‘non-rewarded’ trials, a small reward of 0.05 ml was delivered. Each trial started when the background color on the touch screen changed from black to gray. To initiate the trial, the monkey was required to place his hand on an immobile, touch-sensitive key. Presentation of the gray background was followed by presentation of an ocular fixation spot (1.3° visual angle). On each trial, the animal was then required to fixate this spot within 4° for 500 ms. Following 500 ms of central fixation, a first choice cue (‘object’) and overlaid bar stimulus appeared centrally for 500 ms and were followed, after cue-offset, by a 500 ms inter-stimulus interval, which was then followed by a second choice cue and overlaid bar stimulus shown for 500 ms followed by another 500 ms inter-stimulus interval. The reward magnitude cue covered 18.75 percent of the underlying image. The two objects could have the same reward magnitude on a given trial, as determined by random permutation. We used new objects in each session. Following sequential presentation of these individual choice objects and overlaid bar stimuli, the two objects reappeared simultaneously on the left and right side of the monitor (determined pseudorandomly, balanced across trials); importantly, the magnitude-bar stimuli did not reappear. Thus, the separate presentation of the first and second reward-magnitude cue, and their transient presentation during sequential viewing precluded simultaneous magnitude comparison. After 100 ms, the fixation spot disappeared, indicating that the monkey was no longer required to fixate the spot and was allowed to make his choice by fixating the object on the left or right for 500 ms. The monkey was allowed to freely look back and forth between the objects for 2,000 ms and in that period could make a choice at any time by fixating the chosen object for 500 ms. Once the monkey’s choice was registered, the unchosen object disappeared and after a delay of 500 ms, the chosen object also disappeared and a liquid reward was given depending on the scheduled reward probability and magnitude for the chosen option. Reward delivery was followed by a trial-end period of 1,000 – 2,000 ms which ended with extinction of the gray background. The next trial started after an inter-trial interval of 2,000 – 4,000 ms (drawn from a uniform random distribution). A recording session for a given neuron would typically last 150 trials.

Possible errors in performance included failure to make contact with the touch-sensitive key before the trial, key release before saccade choice, failure to fixate a choice object for 500 ms during the choice period, failure to fixate the central fixation spot at trial start or fixation break in the period between initial fixation and disappearance of fixation spot. Errors led to a brief time out (3,000 ms) with a black background and then trial repetition. Task performance was typically interrupted after three consecutive errors. The animals were required to fixate the fixation spot and the objects until the choice targets were presented in left-right arrangement. Fixation was continually monitored by the task program during all of these periods and fixation breaks resulted in an error trial. The animals were required to place their hand on a touch-sensitive key to initiate each trial and keep their hand in place on the key until trial completion.

### Eye data processing

We continuously monitored and recorded the animals’ eye positions using an infrared eye tracking system at 125 Hz (ETL200; ISCAN) that was placed next to the touchscreen. We calibrated the eye tracker before each testing during a fixation task. During recordings, accuracy of calibration of the eye tracker was checked and recalibrated if necessary.

### Using the continuity axiom for subjective value estimation

Expected Utility Theory (EUT) proposes four axioms that determine the maximization of utility (von Neumann and Morgenstern, 1944). Compliance with completeness (axiom I) and transitivity (axiom II) is necessary for consistently ranking all choice options. Compliance with continuity (axiom III) demonstrates that choices reflect a meaningful representation of numerical utility: the continuity axiom implies the existence of a value function. The independence axiom (axiom IV) defines the computation of expected utility from reward magnitudes and their probabilities. The four axioms of EUT define necessary and sufficient conditions for choices to be described by the maximization of subjective economic value: if the axioms are fulfilled, a subjective economic value can be computed from the reward components (magnitude and probability) for each choice option, and the decision maker behaves as if choosing the highest valued option. Violations of the independence axiom, reported in both human and animal subjects (Allais, 1953; Kagel et al., 1990; Ferrari-Toniolo et al., 2022), are accounted for by non-expected utility theories, such as prospect theory (PT) (Kahneman and Tversky, 1979), which required a weakened form of the independence axiom while retaining the continuity axiom requirement.

The continuity axiom can be formally stated as:

(Eq. 1) ∀A≻B≻C,∃!pA∈(0,1)suchthat(pA⋅A+(1−pA)⋅C)∼B

where A, B, C are gambles, “≻” is the preference relation, and “~” the indifference relation. In words, compliance with the continuity axiom requires the existence of a reward probability pA at which choice indifference occurs between a fixed gamble B and a variable gamble AC = (pA · A + (1 – pA) · C). The variable gamble AC consists of a probabilistic combination of a higher valued gamble A (with probability pA) and a lower valued gamble C (with probability 1 - pA) (Eq. 1).

In both choice tasks used in the current study, the two offered choice options conformed with the continuity axiom definition. In the behavioral choice task (not used during neurophysiological recordings), we defined sure rewards (p = 1) as options A, B and C, resulting in binary choices between a fixed safe option (B) and a gamble with variable probability and fixed magnitudes (corresponding to the AC option in Eq. 1). In the choice task used for neurophysiological recordings, the B option was also a gamble, resulting in choices between a fixed gamble and a gamble with varying probability. The continuity axiom, with an extended testing scheme encompassing a broad set of reward magnitude and probability levels, constituted the foundation of the current study.

### Behavioral indifference points

The behavioral indifference point (IPb) represents a utility measure, being a numerical quantity associated with the subjective evaluation of reward B in relation to outcomes A and C. As a deterministic rule, the axiom assumes constant preferences over time. We interpreted the axiom in a stochastic sense, measuring preferences stochastically over repeated choices, which can potentially fluctuate over time. The IPb was computed according to a standard discrete choice model: we fitted a softmax function to the probability of choosing the AC option and identified the point for which the softmax’ value was 0.5, which corresponded to a 0.5 probability of choosing equally frequently each option, i.e., choice indifference (Figure 1C). The following softmax function was fitted to the choice data through non-linear least squares (Matlab function: nlinfit):

(Eq. 2) PG(pA)=1/(1+exp(−(pA−IPb)/t))

With PG representing the proportion of gamble choices, IPb corresponding to the probability pA resulting in equal preference for the two options, and *t* as softmax “temperature” parameter, representing the steepness of the choice function (steeper for lower τ values), in analogy to the Boltzmann distribution in statistical mechanics. In alternative formulations, the softmax steepness is referred to as β (“inverse temperature” or “precision”), i.e., the reciprocal of the temperature parameter defined here.

### Behavioral indifference map

The indifference curves (ICs) represented in Figure 1H were elicited by fitting a parametric economic value function to the choice data. Following EUT, gamble values can be defined as the product of the corresponding utility and probability components. Using a parametric power function as utility function, we identified the best fitting parameters (softmax parameter, utility parameter) by maximizing the loglikelihood associated with the choice data, as described in detail in our previous behavioral study (Ferrari-Toniolo et al., 2021). To compare the EUT model with a PT-based model, which assumed nonlinear probability weighting, we repeated the fitting procedure using a parametric power function as probability weighting function in addition to the parametric power utility function. As a goodness-of-fit measure, we correlated the probability of choosing the gamble option resulting from measured and modelled choices (Figure 1H inset). We then computed the values corresponding to finely spaced points (resolution 0.01 in both dimensions) within the tested range of reward magnitudes and probabilities. We defined the indifference curves as points with equal value (Matlab *contour* function), corresponding to four safe magnitude levels (0.3, 0.4, 0.5 and 0.6 ml). The curves were plotted for visual comparison with the directly estimated IPs.

### Neurophysiological recordings

Experimental procedures for single-neuron recordings from the amygdala in awake, behaving macaque monkeys followed those described previously (7, 33). A titanium head holder and recording chamber (Gray Matter Research) were fixed to the skull under general anaesthesia and aseptic conditions. The amygdala was located based on bone marks on sagittal radiographs referenced to the stereotaxically implanted chamber (53). We recorded the activity of single amygdala neurons from extracellular positions. We used standard electrophysiological techniques including on-line visualization and threshold discrimination of neuronal impulses on oscilloscopes. We aimed to record representative samples of neurons from the lateral, basolateral, basomedial and centromedial nuclei. We inserted a stainless-steel tube (0.56 mm outer diameter) to guide a single tungsten microelectrode (0.125 mm diameter; 1-to 5-MΩ impedance, FHC Inc.) through the dura. The microelectrode was advanced vertically in the stereotaxic plane with a hydraulic micromanipulator (MO-90; Narishige, Tokyo, Japan). Neuronal signals were amplified, bandpass filtered (300 Hz to 3 kHz), and monitored online with oscilloscopes. Behavioral data, digital signals from an impulse window discriminator, and analogue eye position data were sampled at 2 kHz on a laboratory computer with MATLAB (Mathworks Inc.) code. Analogue impulse waveforms were recorded at 22 kHz with a custom recording system and sorted offline using cluster-cutting and principal component analysis (Offline sorter; Plexon). We used one electrode per recording day and recorded between 1 and 10 neurons per day.

### Neuronal data analysis

We analyzed single-neuron activity by counting neuronal impulses for each neuron on correct trials in fixed time windows relative to different task events focusing on the following non-overlapping task epochs: 500 ms after onset of first cue (probability cue in the non-choice task, first choice option in the choice task), 500 ms after offset of first cue, 500 ms after onset of second cue (magnitude cue in the non-choice task, second choice option in the choice task), 500 ms after offset of second cue. We used fixed-window and sliding-window linear and multi-linear regression analyses to identify neuronal responses related to specific variables. For fixed-window analyses, we first identified task-related object-evoked responses by comparing activity in the cue and post-cue periods to a baseline control period (before appearance of fixation spot) using the Wilcoxon test (*P* < 0.05, Bonferroni-corrected for multiple comparisons). We then used multi-linear regression models to test whether neuronal activities were significantly related to specific task variables (P < 0.05, t-test on regression coefficient). We also used sliding-window multiple regression analyses with a 200-ms window that we moved in steps of 20 ms across each trial (without pre-selecting task-related responses). To determine statistical significance of sliding-regression coefficients, we used a permutation-based approach by performing the sliding-window regression 1,000 times using trial-shuffled data and determining a false-positive rate by counting the number of consecutive sliding-windows in which a regression was significant with *P* < 0.05. We found that less than five percent of neurons with trial-shuffled data showed more than ten consecutive significant analysis windows. Accordingly, we classified a sliding-window analysis as significant if a neuron showed a significant (*P* < 0.05) effect for more than ten consecutive 20-ms windows. Statistical significance of regression coefficients was determined using t-test; all tests performed were two-sided. We performed our regression analysis in the framework of the general linear model (GLM) implemented with the MATLAB function (*glmfit*). We used the following GLM for the non-choice task:

(Eq. 3) $$y=\beta_{0}+\beta_{1}(\;Probability\;)+\beta_{2}(\;Magnitude\;)+\varepsilon$$

with y as the neuronal activity, *Probability* as reward probability and *Magnitude* as reward magnitude. This GLM served to identify probability-coding and magnitude-coding neurons in the non-choice task and to derive regression coefficients for Fig. 3C.

We used the following GLM for analyzing neuronal activity during the first-cue period of the choice task:

(Eq. 4) $$\begin{array}{l} y=\beta_{0}+\beta_{1}(\;Probability\;)+\beta_{2}(\;Magnitude\;)+\beta_{3}(\;ObjectAChoice\;)+\beta_{4}(\;ObjectAFirst\;)+\\ \;\beta_{5}(\;FirstChosen\;)+\varepsilon \end{array}$$

with y as the neuronal activity, *Probability* as reward probability, *Magnitude* as reward magnitude, *ObjectAChoice* as current-trial choice for object A (vs. object B), *ObjectAFirst* indicating whether object A was shown as first (vs. second) object on the current trial, and *FirstChosen* as choice of the first (vs. second) viewed object. This GLM served to identify probability-coding and magnitude-coding neurons in the choice task and to derive regression coefficients for Fig. 5C.

We used the following GLM for analyzing neuronal activity using a sliding window of 200 ms moved in 20-ms steps across the first and second cue periods of the choice task:

(Eq. 5) $$\begin{array}{l} \;y=\beta_{0}+\beta_{1}(\;ProbabilityCue\;1)+\beta_{2}(\;MagnitudeCue\;1)+\beta_{3}(\;ProbabilityCue\;2)+\\ \;\beta_{4}(\;MagnitudeCue\;2)+\beta_{5}(\;ObjectAChoice\;)+\beta_{6}(\;ObjectAFirst\;)+\beta_{7}(\;FirstChosen\;)+\\ \beta_{8}(\;ChosenValue\;)+\varepsilon \end{array}$$

with y as the neuronal activity, *ProbabilityCue*1 and *ProbabilityCue*2 as reward probabilities of cues 1 and 2, *MagnitudeCue*1 and *MagnitudeCue*2 as reward magnitudes of cues 1 and 2, *ObjectAChoice* as current-trial choice for object A (vs. object B), *ObjectAFirst* indicating whether object A was shown as first (vs. second) object on the current trial, and *FirstChosen* as choice of the first (vs. second) viewed object and *ChosenValue* as the value of the chosen option on the current trial. This GLM served to identify probability-coding and magnitude-coding neurons in the choice task and to derive regression coefficients for Fig. 6D, E.

We adapted a method of classification of neuronal value responses based on the angle of regression coefficients (32, 54, 55). In our case, this approach identifies probability-coding and magnitude-coding neurons by testing the statistical significance for a complete model that includes separate regressors for probability and magnitude. Using this method, a neuronal response was categorized as probability-coding or magnitude-coding if it showed a significant overall model fit (P < 0.05, F-test). For responses with significant model fit, we plotted the magnitude of the beta coefficients (standardized slopes) of the two probability regressors on an x-y plane. Following previous studies (32, 54, 55), we divided the coefficient space into eight equally spaced segments of 45° to categorize neuronal responses based on the polar angle in this space of regression coefficients (Fig. 3B). We categorized responses as coding probability if their coefficients fell in the segments pointing toward 0° or 180° or as coding magnitude if their coefficients fell in the segments pointing toward 90° or 270°. We categorized responses as coding both probability and magnitude if their coefficients fell in the segments pointing toward 135° or 315° or in the segments pointing toward 45° or 225°.

### Normalization of population activity

To normalize activity from different amygdala neurons, we subtracted from the trial-specific impulse rate in a given task period the mean impulse rate and divided by its standard deviation (z-score normalization). We also distinguished neurons that showed positive relationships or negative relationships with a given variable, based on the sign of the regression coefficient, and sign-corrected responses with a negative relationship for plotting population activity. Normalized data were used for Fig. 3B-F, Fig. 4.

### Elicitation of neuronal indifference points

Mirroring the behavioral utility measure, we defined a neuronal subjective economic value measure for each tested set of A, B and C options. The neuronal indifference point (IPn) was defined as the gamble probability for which the neuronal response to the safe option matched the response to the gamble option. The IPn was computed as the intersection between two lines: the regression line of the neuronal activity for different gamble probabilities and the line representing the mean response to the safe option. To compute the IPn of individual neurons, responses to the two choice options were directly compared, thus no normalization of neuronal activity was required for this analysis.

### Reconstruction of neuronal recording sites

After data collection was completed, the animals received an overdose of pentobarbital sodium (90 mg/kg iv) and were perfused with 4% paraformaldehyde in 0.1 M phosphate buffer through the left ventricle of the heart. We reconstructed the recording positions of neurons from 50-μm-thick, stereotaxically oriented coronal brain sections stained with cresyl violet based on electrolytic lesions (15–20 μA, 20–60 s, made in one animal) and lesions using cannulas placed to demarcate recording areas, by recording coordinates for single neurons noted during experiments, and in reference to brain structures with well-characterized electrophysiological signatures recorded during experiments (internal and external globus pallidus, substantia innominata) (56). We assigned recorded neurons to amygdala nuclei based on reconstructed recording positions and a stereotaxic atlas (57) at three different anterior-posterior positions (figures in the paper show neuron locations collapsed over anterior-posterior levels).

## Results

### Overview of the study

We investigated the encoding of reward probability, reward magnitude, and related subjective values in primate amygdala neurons across different tasks. We first examined the activity of amygdala neurons recorded in a Pavlovian, non-choice situation in which reward probability and magnitude were cued separately by sequentially presented visual stimuli (Fig. 1). Although the task design was based on a previous study (35), the neuronal data presented here were recorded in conditions designed to test the interchangeability and integration of probability-magnitude combinations and have not been reported before. This study served to investigate whether separate probability and magnitude signals could provide a basis for the encoding of values consistent with the continuity axiom that defines conditions for maximizing reward. We then reanalyzed data from a previous investigation (32), in which amygdala neurons were recorded in a choice task with sequentially presented options varying in reward probability and magnitude. This study served to investigate whether individual amygdala neurons would combine probability and magnitude information into subjective values and related economic choices.

### Design and behavior

We recorded amygdala neurons in a Pavlovian task in which visual conditioned stimuli for reward probability and magnitude were shown sequentially on a given trial (Fig. 1A). This design allowed us to test if amygdala neurons would signal probability and magnitude consistent with principles of the continuity axiom of EUT. Each trial was defined by the combination of probability and magnitude cues (see Methods). These combinations were designed to test neuronal responses to probabilities and magnitudes close to the point of choice-indifference, assessed in a separate choice task (described below). Probability was cued by monochrome sector stimuli, whereas magnitude was cued by a bar stimulus (Fig. 1B). Using a Pavlovian, non-choice task allowed for a clear examination of neuronal probability and magnitude signals, as measured neuronal responses only depended on the cued stimuli but not on additional value-comparison and decision processes.

We used a separate choice task with the same stimuli to determine the monkeys’ subjective indifference points between combinations of probability and magnitude cues. The task was performed during the neuronal-recording period but mostly on separate testing days, in order to maximize trial numbers for each recorded neuron. The monkeys made binary choices between a ‘safe’ option defined by a fixed magnitude and a ‘gamble’ option defined by a combination of varying probability and fixed magnitude (Fig. 1C). By varying the gamble probability across trials and calculating the monkey’s probability of choosing the gamble at different magnitudes of the safe option, we determined the monkey’s indifference point (IP) from psychometric functions (Fig. 1D). This procedure was based on the continuity axiom of EUT, which states that a continuous variation of the reward probability should result in preferences shifting from the safe towards the gamble option, passing through an indifference point. The continuity axiom implies the existence of a continuously varying subjective value function, with the IP representing a quantitative measure of this subjective value. Comparing behaviorally determined indifference points (IPb) with hypothetical objective indifference points (IPev: reward probability for which the gamble’s expected value matched the safe magnitude), identified the monkeys’ risk attitudes. Monkeys were risk seeking if IPb < IPev (preferring the gamble even if its expected value was lower than the magnitude of the safe option) and risk averse if IPb > IPev (preferring the safe option even if its magnitude was lower than the expected value of the gamble).

Figure 1E, F shows choice data and measured indifference points in both monkeys elicited through the procedure described above. In line with the continuity axiom, when increasing the gamble’s reward probability, both animals gradually switched from preferring the safe option to preferring the gamble option. The resulting IPb’s varied when changing the safe and gamble magnitudes, suggesting that the animals considered both reward magnitude and probability information when making choices. The identification of an IPb indicated that magnitude and probability information were integrated into scalar values. The subjective nature of this value quantity was evident in the risk attitudes revealed by the IP measures. We found IPb’s lower than the respective IPev’s, implying a generally risk-seeking attitude in both animals. Our subjective-value measure was stable across testing sessions, consistently reflecting the animal’s risk attitude (Fig. 1G). We elicited IPb’s for different sets of safe and gamble magnitudes, which allowed us to estimate an economic value function from behavioral choices (maximum likelihood procedure, see Methods). The value function was then used to estimate indifference curves (i.e., points with the same subjective value) within a magnitude-probability space (Fig. 1H, Fig. S1). This procedure validated our IPb measures, showing that the elicited indifference curves closely approximated the measured IPb’s across the full range of tested reward magnitude and probability levels (Fig. 1H, inset). As detailed in our previous extended behavioral study (58), this implies that subjective values can be described as the integration of mathematically defined reward magnitude and probability components.

In exploratory analyses, we compared two economic choice models for each animal: a model assuming linear probabilities (as in expected utility theory, EUT), and a model with nonlinear probabilities (as in prospect theory, PT), using information criteria (AIC and BIC) as goodness-of-fit metrics. In monkey A (N = 7,603 trials), the PT model produced lower AIC and BIC values (5,883 and 5,904 respectively) than the EUT model (6,378 and 6,392), indicating a better fit for the model which included the non-linear evaluation of probabilities. In monkey B (N = 1,967 trials) the AIC and BIC values (EUT: 1,543 and 1,554 respectively; PT: 1,544 and 1,561) did not clearly identify a better fitting behavioral model, indicating only a slight advantage for the EUT model. These findings indicate that monkey behaviour was well captured by economic choice models incorporating subjective magnitude representations (utility) and refined by subjective probability representations (weighted probability). Importantly, both EUT and PT models rely on the continuity axiom as a basic requirement for the existence of a scalar subjective value quantity.

Thus, the monkeys’ choices between safe and gamble options reflected the integration of reward probability and magnitude information into scalar subjective values, consistent with principles of the continuity axiom of EUT.

### Neuronal responses to reward magnitude and probability cues

We recorded the activity of 156 amygdala neurons in the Pavlovian task (75/81 neurons in animal A/B) across different amygdala nuclei: lateral (LA, 28 neurons), basolateral (BL, 85 neurons), basomedial (BM, 19 neurons), centromedial (Ce, 24 neurons). During the experiments, we sampled activity from about 300 neurons and typically recorded and saved the activity of those neurons that appeared to respond to any task event during online inspection of several trials. We aimed to identify task-responsive neurons but did not preselect based on particular response characteristics. This procedure resulted in a database of 156 neurons that we recorded and analyzed statistically.

**Figure 2:**
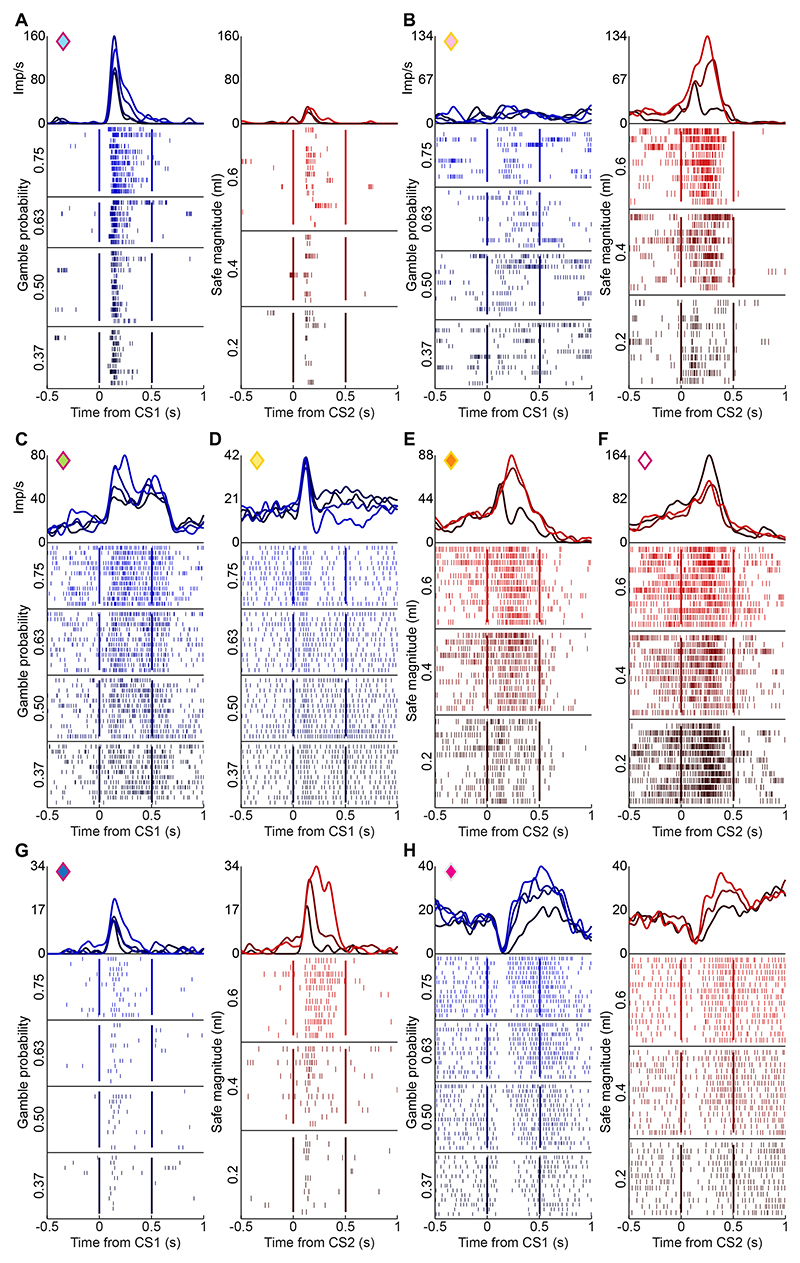
Coding of magnitude and probability in individual amygdala neurons. A, B) Example neurons coding either reward probability (p, panel A) or reward magnitude (m, panel B). Raster plot of action potentials (one trial per row) and spike density function (top; gaussian-convoluted activity rate, 25 ms kernel) for an individual neuron. Left: response to four options’ probabilities (Gp). Right: response to three safe options’ magnitudes (Sm). CS1: first conditioned stimulus, representing probability information; CS2: second conditioned stimulus, representing magnitude information (see Figure 1A, B). Diamond symbols identify each neuron’s anatomical location (see Figure 3A). C-F) Example neurons coding either reward probability (C, D) or reward magnitude (E, F). Neurons responded either proportionally (C, E) or inversely (D, F) to the coded variable (either reward magnitude or probability). G, H) Example neurons coding both reward probability and magnitude.

**Figure 3:**
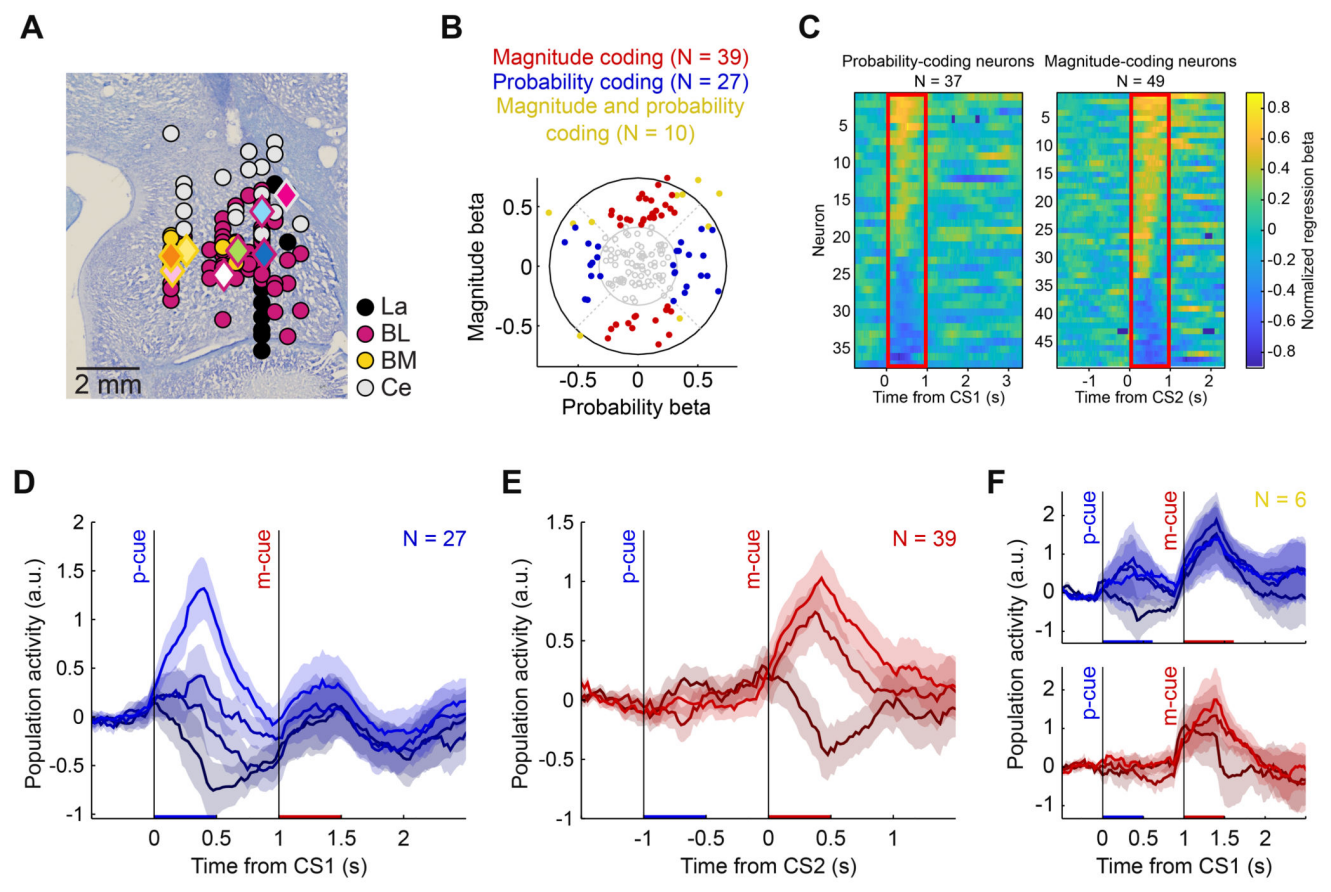
Coding of magnitude and probability in populations of amygdala neurons. A) Histological reconstruction of recorded neurons’ anatomical locations. Dots correspond to individual recorded neuron (collapsed across anterior-posterior axis, fill colors identify the corresponding amygdala nucleus (La: lateral, BL: basolateral, BM: basomedial, Ce: central). Diamond colors represent the example neurons reported in Figure 2 (fill) and identify the amygdala nucleus (outline). B) Coding of magnitude and probability across the neuronal population. Normalized regression coefficients (betas) for magnitude and probability. Each dot represents a neuron with significant magnitude and probability betas (purple), significant magnitude beta only (red) or significant probability beta only (blue). Grey: no significant beta. C) Time course of magnitude and probability coding. Neurons with significant probability-beta only (left) and magnitude-beta only (right) coefficients, sorted by beta values averaged within the 1 s post-cue time window (red rectangle). Both populations included neurons with significant positive and negative betas, corresponding to positive and inverse coding of the corresponding reward variable, respectively. D-F) Population responses. Average normalized population activity (area: SE) across the populations of probability-coding neurons (D) (3 magnitude levels: m = 0.2, 0.4, 0.6 ml), magnitude-coding neurons (E) (4 probability levels; p = 0.37, 0.50, 0.63, 0.75) and simultaneous magnitude-probability (same-sign) coding neurons (F). Neurons showing inverse coding of either magnitude or probability were rectified and included in the analysis.

Figure 2. illustrates the different observed response profiles of amygdala neurons, by showing the responses of eight amygdala neurons to cues indicating different levels of reward probability and reward magnitude. The neuron in Fig. 2A was recorded in the BL nucleus (cf. Fig. 3A) and showed a selective response to the probability cues that increased monotonically with the indicated reward probability; the neuron showed little response to reward-magnitude cues. By contrast, the neuron in Fig. 2B, recorded in the BL nucleus, showed the opposite activity pattern by responding with monotonically increasing activity to different cued reward-magnitude levels but showing little response to probability cues. Figure 2C-F illustrates further examples of probability-selective (Fig. 2C, D) and magnitude-selective neurons (Fig. 2E, F) with activities that either monotonically increased (Fig. 2C, E) or decreased (Fig. 2D, F) with increases in the encoded variable. Figure 2G, H shows two neurons with activity that increased with both reward probability and reward magnitude levels. Thus, individual amygdala neurons showed different types of activity patterns that coded the cued reward probability, reward magnitude or both variables. We next quantified the presence of these different neuron types in the population of recorded amygdala neurons.

Among 156 recorded amygdala neurons (Fig. 3A), 27 neurons (17%, 15/12 animal A/B) were classified as coding reward probability, 39 neurons (25%, 19/20 animal A/B) were classified as coding reward magnitude and 10 neurons (6%, 4/6 animal A/B) were classified as coding both probability and magnitude (Fig. 3B). Neurons were classified using a multiple-regression analysis based on the angle of probability- and magnitude-coefficients (32, 54, 55, 59). Amygdala neurons coding reward probability and/or reward magnitude were found in all sampled amygdala nuclei (Fig. 3A, probability coding in LA/BL/BM/Ce: 4/15/4/4; magnitude coding in LA/BL/BM/Ce: 8/20/6/4; probability and magnitude coding in LA/BL/BM/Ce: 2/7/0/1). Responses of probability-coding neurons and magnitude-coding neurons were typically phasic, time-locked to the onset of the relevant cue, and showed positive and negative coding schemes in approximately similar proportions (Fig. 3C; probability-coding, positive/negative: 16/11; magnitude-coding, positive/negative: 26/13). The graded responses to different probability and/or magnitude levels were prominent in the aggregated population activity (Fig. 3D-F).

We examined if anticipatory licking in the cue phases influenced neuronal coding of probability and magnitude, focusing on animal B (animal A did not show anticipatory licking during the cue phases). Among 48 neurons with at least 31 trials on which licking occurred (mean 54.4 ± 8.0 sd), 6 coded probability (13%) and 9 coded magnitude (19%). When a covariate for duration of anticipatory licking was included in the regression, 5 neurons coded probability and 8 neurons coded magnitude. Thus, licking had little impact on neuronal coding of probability and magnitude.

Thus, different groups of amygdala neurons coded the cued reward probability or reward magnitude and thereby signalled the two distinct components of subjective values in our task. We next examined whether these responses were consistent with key notions of the continuity axiom.

### Neuronal indifference points

Using the continuity axiom as a guiding principle, we tested whether amygdala neurons encoded reward information consistent with mathematically defined subjective values. To this aim, we analyzed the neuronal responses to different safe and gamble options for which we also measured the monkey’s behavioral preferences. Importantly, given the subjectivity of economic value measures, we only directly compared behavioral and neurophysiological data from the same animal. Neuronal responses representing subjective values should reflect the monkey’s preferences, with higher sign-corrected response to a preferred option compared to the non-preferred option, and equal response for equally preferred options. In our continuity scheme, this implies that, after sign correction, the gamble response, compared to the safe response, should be lower for non-preferred gambles, higher for preferred gambles and equal for equally chosen safe and gamble options.

**Figure 4:**
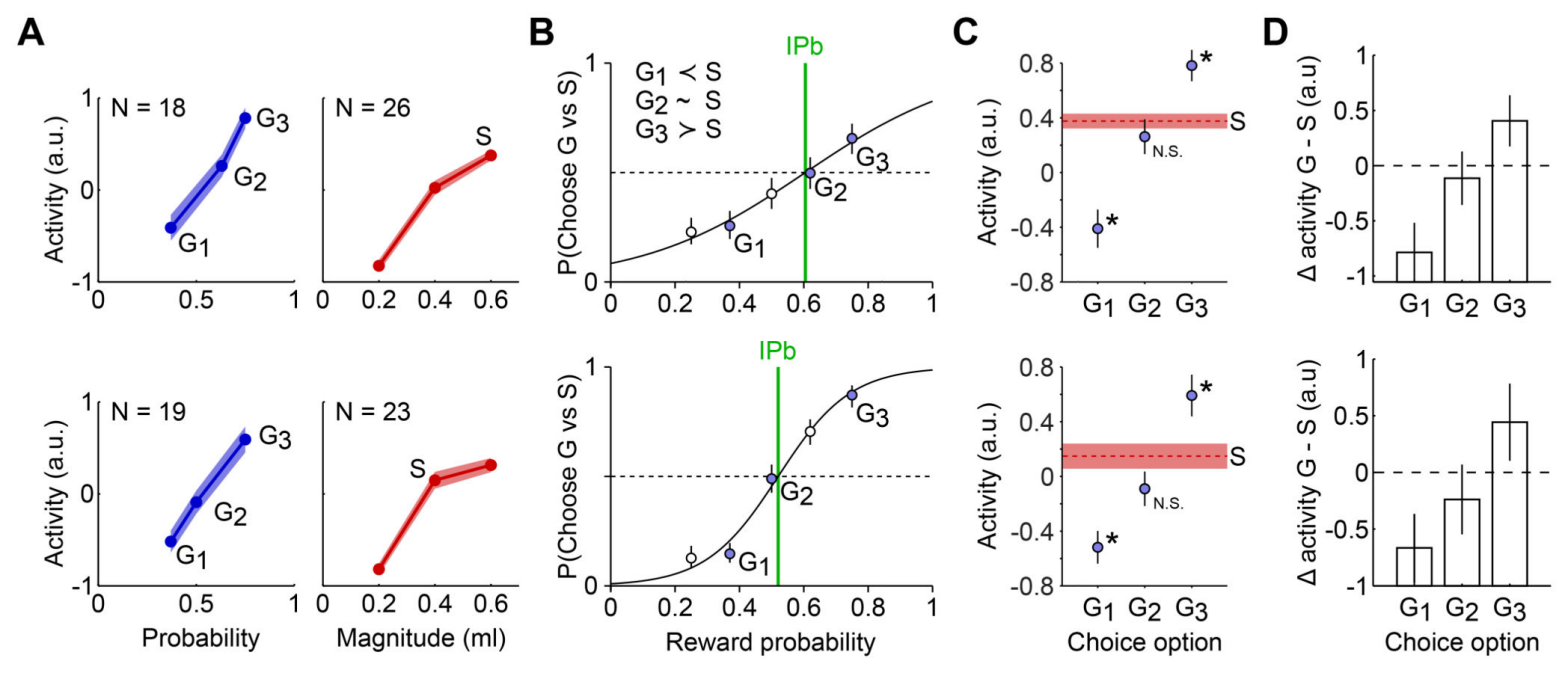
Match between behavioral and neuronal indifference points. A) Response to three gamble options (left) and three safe options (right) for the neuronal populations encoding reward probability and magnitude (left and right, respectively). Activity was sign-corrected after normalization. S: safe option selected for subsequent data analysis (corresponding to behavioral choice indifference between S and G_2_). Area: SE. Data from monkey A (top) and monkey B (bottom). B) Behavioral preferences. Probability of choosing each gamble option (filled circles: G1, G2 and G3) against a fixed safe option (S), as a function of gamble probability. Monkeys had well defined preferences between each gamble and the safe option S: G_1_ non preferred, G_2_ indifferent, G_3_ preferred. Open circles: other tested gambles. Other conventions and symbols as in Figure 1E-F. C) Population response to the selected options. Responses to the safe S option (dashed line: mean, area: SE) and to each of the three gambles (circles) were compatible with the behavioral preferences: response to S was significantly different from response to G_1_ and G_3_, while being not significantly different (N.S.) for G_2_. Statistical p-values (unpaired t-test) for G_1_, G_2_ or G_3_, respectively: 1.0E-3, 0.35, 5.2e-7 (monkey A, top), 1.2e-2, 0.13, 5.9e-5 (monkey B, bottom). Asterisk, P < 0.05. D) Difference (Δ) in population activity between gamble and safe options. Compatible with a subjective-value coding signal, the activity difference was negative for G_1_, positive for G_3_ and non-significantly different from zero for G_2_. Bars: 95% confidence intervals.

Separately for the populations of probability-coding and magnitude-coding amygdala neurons, we pooled the neuronal responses to different gamble probabilities and safe-option magnitudes, respectively (Fig. 4A). To assess value coding in the neuronal population, we then selected three gamble options (G1, G2, G3) which were respectively behaviorally non-preferred, equally preferred, and preferred in relation to a fixed safe option (S) (Fig. 4B). This was done separately for each monkey, to account for their specific subjective evaluations. We found that the average responses to the selected safe and gamble options reflected the behavioral preferences. Compared with the responses to the S option, responses to G1 were significantly lower, responses to G2 were non-significantly different, and responses to G3 were significantly higher (Fig. 4C, D). This result was confirmed in both animals, reflecting their individual preferences.

Notably, this pattern was not predetermined by neuron selection or our analyses approach. Neurons were selected for coding probability or magnitude but this did not guarantee that responses to the intermediate gamble matched the behavioral indifference point: swapping safe options within animal or between animals reduced neuronal-behavioral matches (Fig. S2A-D). Further, ranking of gamble options was robust to changes in normalization procedures (Fig. S2E, F).

These data show that the reward probability and magnitude components, separately coded by different groups of amygdala neurons, reflect individual preferences, suggesting the coding of subjective values for gamble and safe choice options.

### Coding of reward magnitude and probability during choice

**Figure 5:**
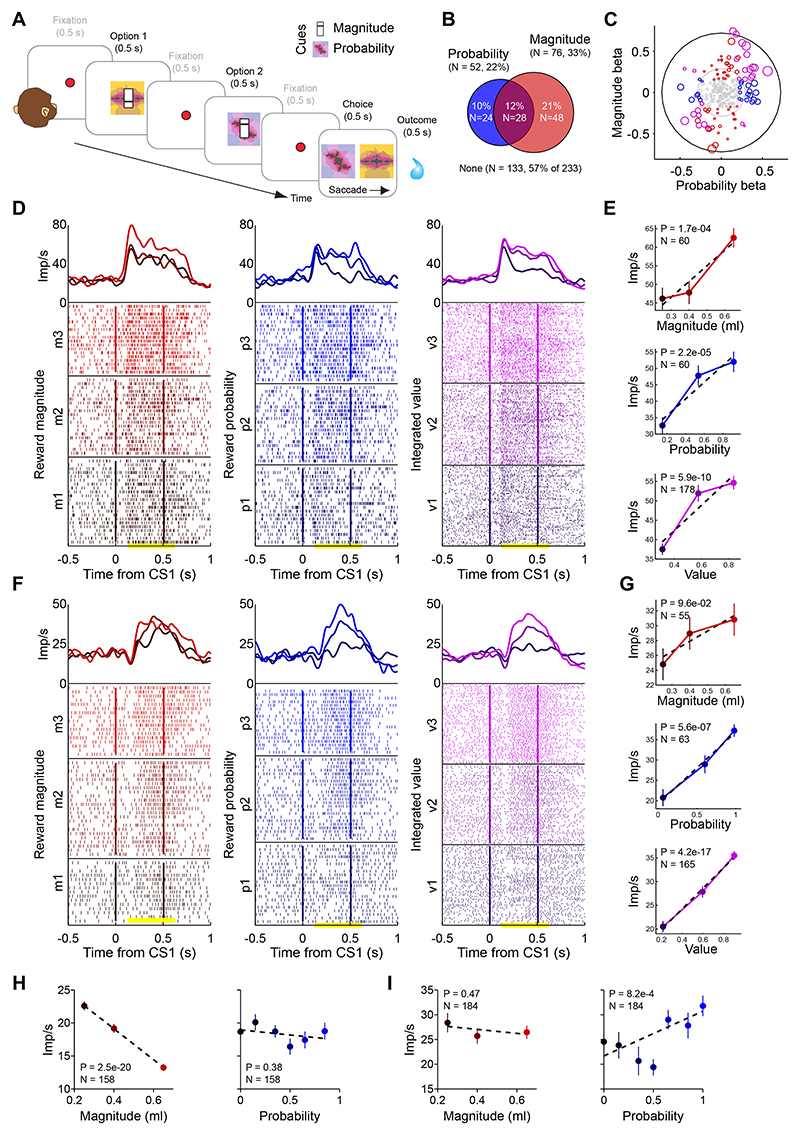
Integration of magnitude and probability information in individual neurons during choice. A) Choice task. Monkeys choose between sequentially viewed options based on randomly varying reward probability and magnitude combinations. Inset: Reward value derives from block-wise changing, uncued reward probabilities and trial-specific, transiently cued magnitudes. B) Venn diagram representing neurons with significant regression coefficients for reward magnitude (red), probability (blue) or both (purple). Data from both monkeys. C) Regression beta coefficients for reward magnitude and probability. Colors identify significant betas for reward magnitude (red), probability (blue), both (purple) or none (gray). Circle size is proportional to the beta associated with the coding of integrated value. D) Response of an example neuron to the cue representing one choice option (raster plot and spike density function). Left: varying magnitude (fixed probability); center: varying probability (fixed magnitude); right: varying integrated value (all magnitude and probability levels). The option’s reward magnitude was explicitly cued (three levels), while the probability had to be inferred from experienced outcomes. We estimated the learned probability through a reinforcement learning model, which also output a trial-by-trial estimate of the option’s integrated subjective value. The distribution of probabilities and subjective values were divided into terciles for data analysis, resulting in the three levels reported on the vertical axes (m_i_, p_i_, v_i_; i=1, 2, 3). The intermediate probability tercile (p_2_) and the second magnitude level (m_2_) were used as fixed levels in the magnitude and probability plots, respectively. The neuron’s responses significantly correlated with the cue’s reward magnitude, probability and integrated subjective value. E) Single neuron’s mean response rate ± SE (time analysis window: yellow line in panel D) to different magnitudes (top), probabilities (middle), and subjective values (bottom). Dashed line: linear regression. P: p-value from correlation analysis. F, G) Response of a different example neuron encoding a cue’s reward magnitude, probability and integrated subjective value. Conventions as in panels D and E. H-I) Example neurons encoding exclusively reward magnitude (H) or reward probability (I).

So far, we examined the neuronal representation of reward magnitude and probability, separately cued in a non-choice, Pavlovian task. We next investigated the integrated coding of these fundamental reward variables in a separate choice task (Fig. 5A). This approach allowed us to identify neuronal signals reflecting values that matched the economic values defined by each monkey’s behavioral ones, resulting from the integration of reward magnitude and probability information, and their translation to choices.

We recorded 233 amygdala neurons (180/53 in monkeys A/B) while the same two monkeys tested in the Pavlovian task performed a reward-based decision task (32). In each trial, two choice options were presented sequentially in two successive cue periods, followed by a side-by-side presentation of the same pair of options. The animals were required to integrate two value sources: tracking the slowly varying reward probabilities associated with each cue (different visual images), and combining them with trial-specific magnitudes cued by explicit bar stimuli (Fig. 5A, inset). Thus, each option’s reward magnitude was explicitly cued (three possible levels), while the probability had to be learned from experienced rewards. Magnitudes were cued transiently during sequential presentation (but not during the choice epoch) to encourage valuation and decision-making during sequential viewing. The monkey made a choice through a saccade towards one of the cues, receiving the corresponding reward outcome. All cue presentations (duration: 0.5 s) were separated by central fixation periods of 0.5 s (Fig. 5A).

We previously described that the monkeys’ choices in this task reflected the integration of reward probabilities and magnitudes for sequentially viewed options and that amygdala neurons encoded subjective values and related choices that reflected the integrated probability and magnitude information (32). Here, we analyzed the neuronal responses in relation to the separately varying magnitude and probability value components to test whether neuronal value signals complied with assumptions of the continuity axiom. In the first cue period, 24/233 amygdala neurons (10%) encoded reward probability but not magnitude, 48 neurons (21%) encoded reward magnitude but not probability, and 28 neurons (12%) were classified as encoding both probability and magnitude (Fig. 5B, C). The corresponding number of neurons encoding probability, magnitude or both probability and magnitude for the first post-cue delay period were 8 (3%, 7/1 in animal A/B), 26 (11%, 22/4 in animal A/B), 42 (18%, 35/7 in animal A/B); for the second cue period were 31 (13%, 26/5 in animal A/B), 29 (12%), 57 (24%, 47/10 in animal A/B), and for the second post-cue delay period were 7 (3%, 5/2 in animal A/B), 28 (12%, 21/7 in animal A/B), 38 (16%, 32/6 in animal A/B).

We also tested for multiplicative encoding of probability and magnitude by adding an interaction term to the regression model. In the first cue period, 20 neurons (9%) showed a significant interaction between probability and magnitude. Significant interaction effects in the first post-cue delay period, second cue period, and second post-cue delay period were found in 20 (30%), 22 (9%) and 21 (9%) neurons, respectively. Inclusion of the interaction term in the regression led to improved model fit (higher adjusted R^2^) in 44% of tested neuronal responses across the four time periods but did not substantially change the number of identified probability-coding neurons (23, 32, 20, 14 in the four periods) or magnitude-coding neurons (59, 44, 62, 36 in the four periods).

The activity of the two amygdala neurons in Fig. 5D, E and Fig. 5F, G increased with both higher probability and higher magnitude levels at the first cue and accordingly were best explained in terms of integrated value coding. By contrast, the amygdala neuron in Fig. 5H encoded only the reward magnitude but not probability, while the amygdala neuron in Fig. 5I encoded only the reward probability but not magnitude.

Thus, in the value-based choice task, different amygdala neurons either integrated the reward probability and magnitude components into subjective value or selectively encoded one of these two value components. We next investigated whether these neurons’ activities were consistent with principles of the continuity axiom.

### Integration of magnitude and probability information during choice

We next compared indifference points estimated from behavioral and neuronal data in the choice task, as the continuity axiom implies equal choice of different magnitude-probability combinations. Thus, we tested whether neuronal responses reflected values consistent with choice behavior (i.e., ‘behavior-matching’ values), rather than objective expected value or other combinations of the reward variables.

**Figure 6:**
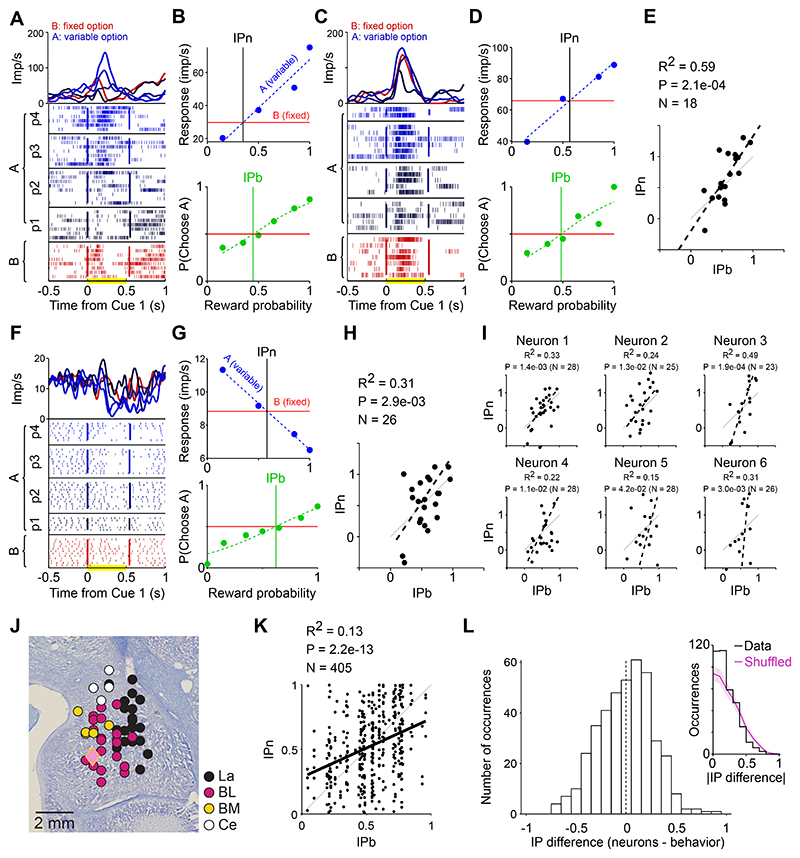
Behavior-matching neuronal subjective values compatible with the integration of magnitude and probability information. A) Neuronal responses of an individual neuron (raster plot and spike density function) to cues representing either a fixed choice option B (red; magnitude = 0.40 ml, probability = 0.15) or a variable choice option A (blue) with fixed magnitude (0.25 ml) and variable probability p_i_ (0.15, 0.50, 0.85 or 1.00). Yellow marking: analysis period. B) Neuronal (top) and behavioral (bottom) indifference points. Neuronal indifference point (IPn) was computed as the intersection between the linear fit (dashed line) of the mean responses to the variable option A (blue dots) and the mean response to the fixed B option (red line). Behavioral indifference point (IPb) was estimated via softmax fit to the probability of choosing the variable option A over the fixed option B (behavioral data from all sessions). C-D) Response of the same neuron as in panels A-B, for a different set of choice options (fixed B magnitude: 0.40 ml, probability: 0.85; variable option A magnitude: 0.65 ml). E) Correlation between the neuronal and behavioral indifference points (IPb and IPn respectively) across all tested choice options for example neuron for 18 different sets of choice options (same neuron as in panels A-D). F) Neuronal responses of a different neuron to cues representing fixed choice option B (red; magnitude = 0.40 ml, probability = 0.15) or variable choice option A (blue) with fixed magnitude (0.25 ml) and variable probability p_i_ (0.15, 0.50, 0.85 or 1.00). G) Neuronal (top) and behavioral (bottom) indifference points for the neuron in F. H) Correlation between the neuronal and behavioral indifference points across all tested choice options for example neuron for 26 different sets of choice options (same neuron as in panels F-G). I) Correlation between the neuronal and behavioral indifference points for six further individual neurons. J) Histological reconstruction of the anatomical locations of the neurons modulated by both reward magnitude and probability in any task epoch (N = 61, collapsed across anterior-posterior axis). We considered four 0.5 s task epochs: CS1, post-CS1, CS2 and post-CS2. Diamond symbol: example neuron shown in panels A to E. K) Correlation between neuronal and behavioral IP measures, across all neurons with significant IPn-IPb correlation and all tested combinations of choice options (N = 405). L) Distribution of differences between neuronal and behavioral indifference points (IPn – IPb) across the population of neurons with significant IPn-IPb correlation (N = 32), for all tested combinations of choice options (N = 405). Inset: distribution of absolute differences (|IPn - IPb|) computed from the collected data and from randomly shuffled data (shaded area: SD across 2,000 repetitions). The higher occurrence of lower differences in the original data compared to the randomly shuffled data (significant difference, Kolmogorov-Smirnov test, P = 8.5e-36, N = 405) supported the coding of behavioral-matching indifference points in amygdala neurons.

As with the Pavlovian task, this procedure was based on the continuity axiom of EUT, with one option (A) varying in probability (across blocks of trials), while the other option (B) was fixed. We analyzed the neuronal responses to options A or B in the first cue period (Fig. 6A, Eq. 4). This analysis period was selected in order to examine potential value-coding prior to value comparison and decision-making, which happened at the second cue period. A behavior-matching subjective-value coding neuron would respond equally to the A and B options that were equally chosen by the monkey. To test this notion, we calculated a neuronal indifference point (IPn) as the intersection between a linear fit of the mean responses to the variable option A and the mean response to the fixed B option. We compared the resulting IPn (Fig. 6B, top) with the corresponding behavioral IPb (Fig. 6B, bottom). Importantly, neurons used for this analysis were not preselected for coding subjective value, but only for coding both magnitude and probability.

In one example neuron (Fig. 6A-D), behavioral and neuronal indifference points appeared similar within the same set of A and B options (Fig. 6B, C). When varying the A and B options values, both IPb and IPn values appeared higher than those estimated for the first set of options (Fig. 6D). To statistically evaluate the neuronal-behavioral IP relation, we performed a correlation analysis across different A and B option sets (N = 18 option sets for this neuron). We found a significant correlation between IPb and IPn measures, suggesting that this example neuron encoded behavior-matching subjective values across the experienced reward magnitudes and probabilities (Fig. 6E). Detailed data from another example neuron are shown in Fig. 6F-H, and behavioral-neuronal matching indifference points for six further neurons are shown in Fig. 6I.

In the population of amygdala neurons that responded significantly to both magnitude and probability (N = 28), 16 neurons showed a significant IPb-IPn correlation, while the average difference between IPb and IPn across neurons and IP measures (N = 170) was not significantly different from zero (t-test, P = 0.72, N = 170, mean and SD: 0.0081 ± 0.29), suggesting coherent subjective-value coding in the population of amygdala neurons sensitive to both reward magnitude and probability. These results were confirmed when including three further task epochs of 0.5 s each (post-CS1, CS2 and post-CS2). We found N = 61 neurons responding significantly to both magnitude and probability in at least one of the four epochs. Among these neurons, which were distributed across different amygdala nuclei (Fig. 6J), we found 32 correlated neuronal and behavioral IP measures out of 74 total tests. A significant IPb-IPn correlation was confirmed in the full set of neurons and IP measures (N = 405) (Fig. 6K). The average difference between IPb and IPn was not significantly different from zero (t-test, P = 0.81, N = 405, mean and SD: -0.0032 ± 0.27) and resulted in a better neuronal-behavioral match than randomly shuffled data (Fig. 6L).

These data show that a population of amygdala neurons represented the integration of reward magnitude and probability information during the choice task at the level of individual neurons by encoding a subjective-value measure that reflected the monkey’s preferences.

### Value-to-choice coding transitions in amygdala neurons

**Figure 7:**
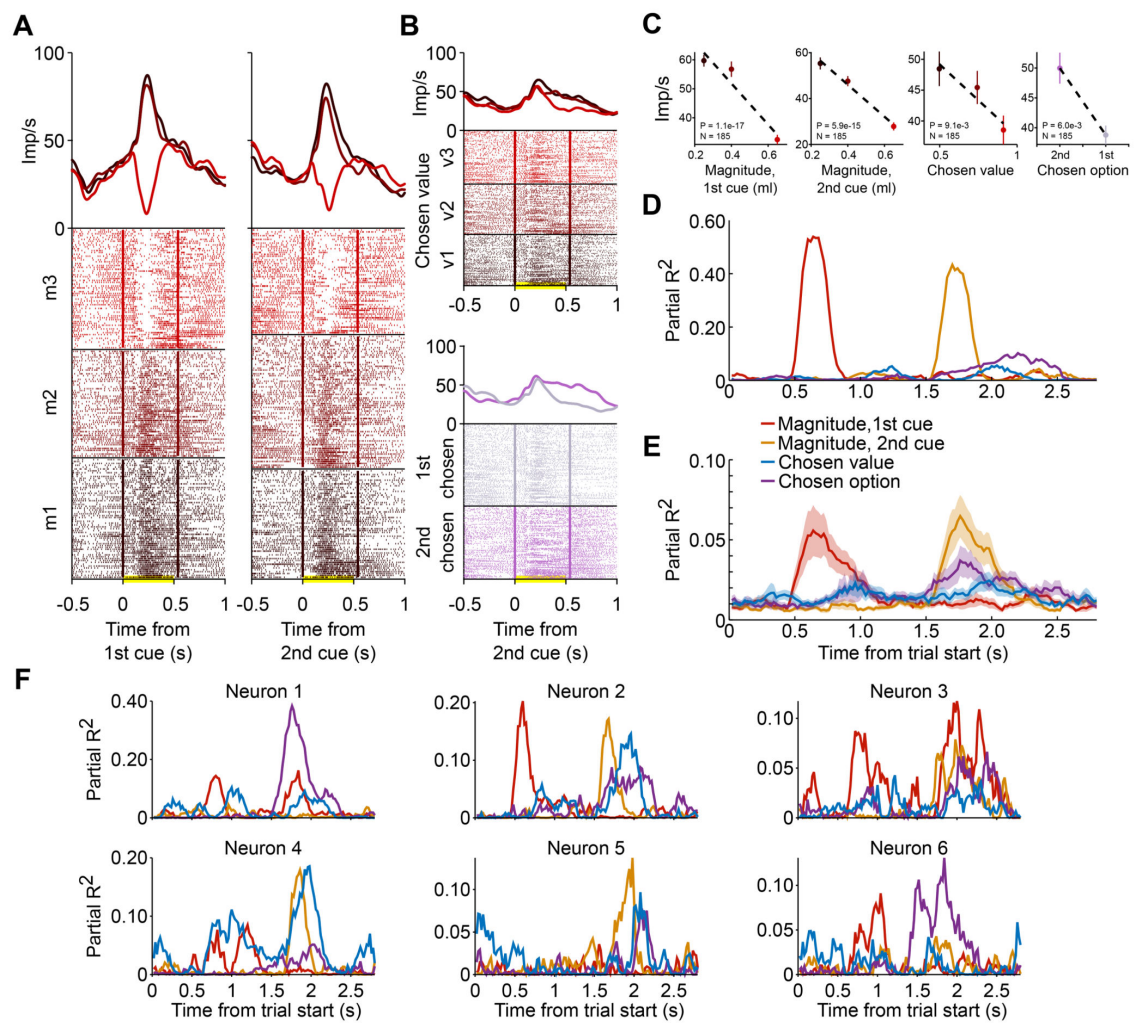
Neuronal value-to-choice transitions during decisions. A) Activity of one amygdala neuron coding the magnitude of the first (left) and second (right) choice option. B) Activity of the same neurons as in A) transitioned to coding the chosen value (top) and choice (bottom) on the current trial (right). C) Neuron’s mean response rate ± SE (time analysis window: yellow line in panels A) and B) to different magnitudes, chosen value, and choice. Dashed line: linear regression. P: p-value from correlation analysis. D) Coefficients of partial determination (partial R^2^) from sliding-window multiple regression analysis of the neuron’s activity, showing periods of significant coding for magnitudes, chosen value and choice. E) Mean coefficients of partial determination (± SE) for 55 amygdala neurons identified as coding the monkeys’ choices in a sliding-window multiple regression analysis, showing population-coding of magnitudes of the first and second option, chosen value and choice. F) Six further example neurons showing value-to-choice transitions. Neuron 1: coding first magnitude, choice and chosen value; Neuron 2: coding all variables; Neuron 3: coding first and second magnitude and choice; Neuron 4: coding all variables; Neuron 5: coding second magnitude, choice, and chosen value; Neuron 6: coding first magnitude and choice.

We next examined whether amygdala neurons that encoded value information were directly implicated in decision-making processes. To this aim, we investigated the coding of different variables underlying the choice process. Focusing on reward magnitude, chosen value and chosen option, we quantified how well these variables were represented in individual neurons over different time windows. We previously showed (7, 32, 33, 59) that amygdala neurons implicated in economic decision-making exhibit dynamic coding patterns that transition from coding value (i.e., decision inputs) to coding choice (i.e., decision outputs). The amygdala neuron in Fig. 7A, B exhibited a value-to-choice transition by initially signaling the magnitude of the first cue (Fig. 7A) and then signaling the magnitude of the second cue, the monkey’s forthcoming choice for the first or second option on a given trial, as well as the related chosen value (Fig. 7B). These dynamic coding transitions were evident in the regressions of the neuron’s activity on the different variables (Fig. 7C) and in phasic peaks of the coefficients of partial determination for each variable (Fig. 7D, Eq. 5). Dynamic value-to-choice transitions were expressed prominently in amygdala neurons that encoded the monkeys’ choices (N = 55, Eq. 5, Fig. 7E): in this subgroup of neurons, phasic signals related to the magnitude of the first and second option often preceded signals related to the monkeys’ trial-specific choice and to the chosen value. Among 55 choice-coding neurons, 10 neurons (18%) also coded the magnitude of the first and second cue and chosen value, and 11 neurons (20%) coded the magnitude of the first and second cue without coding chosen value.

Thus, the activity of amygdala neurons with phasic responses to reward-magnitude cues frequently transitioned to coding the monkeys’ choices and chosen values, consistent with involvement of these neurons in several stages of economic decision processes.

## Discussion

These results show that primate amygdala neurons encode the basic components of economic value, reward magnitude and probability, in compliance with formal tenets of economic theory that deal with reward maximization. According to the continuity axiom of EUT, a decision-maker should be indifferent between the intermediate of three subjectively ranked gambles and a probabilistic combination of the other two. The compliance of the monkeys’ behavioral choices with the continuity axiom is consistent with the integration of reward magnitude and probability into a scalar value quantity. This integration represents the continuous tradeoff between reward components (a decrease in magnitude is compensated by an increase in probability, and vice versa), as shown in the behavioral indifference map (Fig. 1). The robustness of these conclusions relied on testing the axiomatic rule with a sufficient range of magnitude and probability levels. Although we previously showed that amygdala neurons carry signals for probability and magnitude (35), their integration and interchangeability for formally defined values had remained untested. When tested in neurons, compliance with the continuity axiom reflected the integration of magnitude and probability information into a value signal. By itself, this result does not demonstrate encoding of subjective values, as it may simply reflect the monotonic neuronal responses to both reward magnitudes and probabilities (Fig. 2, 3, 5). However, by comparing the behavioral and neuronal subjective-value measures (i.e., the indifference points based on the continuity axiom), we ensured that the neuronal value code reflected the individual animal’s subjective evaluation of the choice options, in both no-choice (Fig. 4) and choice situations (Fig. 6). Single-cell and population signals translating these subjective evaluations into choice predictions and chosen-value signals (Fig. 7) demonstrate that amygdala neurons encode key variables underlying economic choice in a manner suitable for maximizing reward.

Although previous studies identified neurons encoding subjective values in the amygdala (7, 20, 31–33, 37), the orbitofrontal cortex (9, 10, 12, 14, 15, 17), and other brain structures (1–3, 13, 16, 20, 43, 55, 59–62), only two previous studies examined compliance of neuronal value signals with the continuity axiom of EUT (12, 42). The present results contribute to the emerging picture of a neuronal value-based choice mechanism compatible with axiomatic economic principles. Specifically, the amygdala may participate together with the orbitofrontal cortex and the midbrain dopamine area in a distributed, formally coherent valuation system.

In our non-choice task with separately cued reward magnitude and probability components, the majority of amygdala neurons encoded only one of the two reward components. This might reflect subjective value coding for specific categories of choice options (in this case, safe and gamble options). Alternatively, it could reflect the coding of only one reward component, a feature that would be required for integrating magnitudes and probabilities into a single scalar quantity. As we only used one reward magnitude for the gamble option in the no-choice task, further tests are necessary to discriminate between these two hypotheses, by simultaneously varying both reward magnitude and probability of the gamble options. Another possibility is that amygdala neurons encode reward and decision-variables in a context-sensitive manner. For example, previous studies showed that neuronal coding of valuation and decision variables in a sequential reward-saving task was for many amygdala neurons specific to free-choice (compared to forced-choice) contexts (7, 36, 37). Our recent study on amygdala neurons in a non-choice task, using different task parameters, showed that few neurons integrated probability and magnitude into expected value without explicit requirement for decision-making (35). Our present results on amygdala neurons in non-choice and choice situations show that amygdala neurons do integrate both value components into subjective value signals in choice situations. Thus, neuronal integration of reward parameters into decision variables in the amygdala appears sensitive to context, i.e., the presence of decision-making requirements.

EUT defines the exact computation for combining reward components into scalar subjective values. According to EUT, the subjective value coincides with the utility associated to a choice outcome weighted by its probability of occurrence. The fourth axiom of EUT, the independence axiom, demonstrates this multiplicative form of value computation. Although this additional axiom is often violated in humans and monkeys (45, 47, 52), a simple modification of the value computation mechanism employing subjectively weighted probabilities was proposed to account for these violations. Probability weighting was formalized in prospect theory as a nonlinear transformation of gamble probabilities (48, 63), and its neuronal representation has been isolated in the orbitofrontal cortex, striatum and dopaminergic midbrain (12–16, 42, 43). In the experimental conditions reported here, the behavior was well captured by EUT, with improved fit for the prospect theory model only in one animal. Since our experiment was not designed to explicitly elicit and test nonlinear probabilities, which would require a broad set of probability levels, we did not assess whether amygdala neurons complied with a prospect theory-based representation of reward components. It will be important in future work to expand our approach to conclusively test whether amygdala neurons comply with specific elements of prospect theory, such as nonlinear probability weighting.

How could the presently reported neurons coding subjective values contribute to broader amygdala functions? The behavioral relevance of value-coding neurons in amygdala may be reflected by effects of amygdala damage: in monkeys and humans, amygdala lesions alter reward-guided behaviors and economic decisions (41, 64–71). Consistently, human neuroimaging studies find subjective value signals in the amygdala (38–41). In the present study, histological reconstructions showed that value-coding neurons were prevalent across the amygdala subdivisions we recorded from, including primarily the lateral and basolateral nuclei, but also (with fewer sampled neurons) in basomedial and central nuclei. As these nuclei differ in input and output connections, local circuit-designs and functions (72–81), value-coding neurons in each nucleus might contribute to different functions. The lateral nucleus might constitute the primary site for associating value information with visual cues (33, 77). The present results suggest that amygdala neurons encode values—including at this early processing stage—in a manner that reflects the subjective tradeoff between reward components. By contrast, neurons in the basolateral amygdala have been more directly implicated in decision (32) and in integrating magnitude and probability information into risk (35). Thus, the presently reported value-coding neurons in basolateral amygdala might contribute to local decision computations and decision processes in other brain structures to which this nucleus projects, including the orbitofrontal cortex and striatum. Finally, value-coding neurons in central nucleus—the amygdala’s principal output structure to autonomic, endocrine, and attentional effector systems (77, 78)—may play roles in translating value into physiological and emotional responses (82, 83).

The amygdala is implicated in a variety of psychiatric and behavioral conditions in which reward valuation, decision-making and mental health are affected (84–89). Our findings that amygdala value computations can be formalized using axioms of economic theory advances our understanding of the amygdala’s role in neuropsychiatric conditions. For example, reduced amygdala value sensitivity or deficient neuronal integration may contribute to maladaptive or unstable preferences.

In conclusion, our results demonstrate compliance with the continuity axiom of EUT in both behavior and neuronal responses and behavioral-neuronal subjective value measures in the amygdala. These findings identify amygdala neurons as substrates for encoding behavior-matching subjective values according to the continuity axiom of EUT, and for translating these values into economic choices.

## Supplementary Material

Supplementary Material

## Figures and Tables

**Figure:**
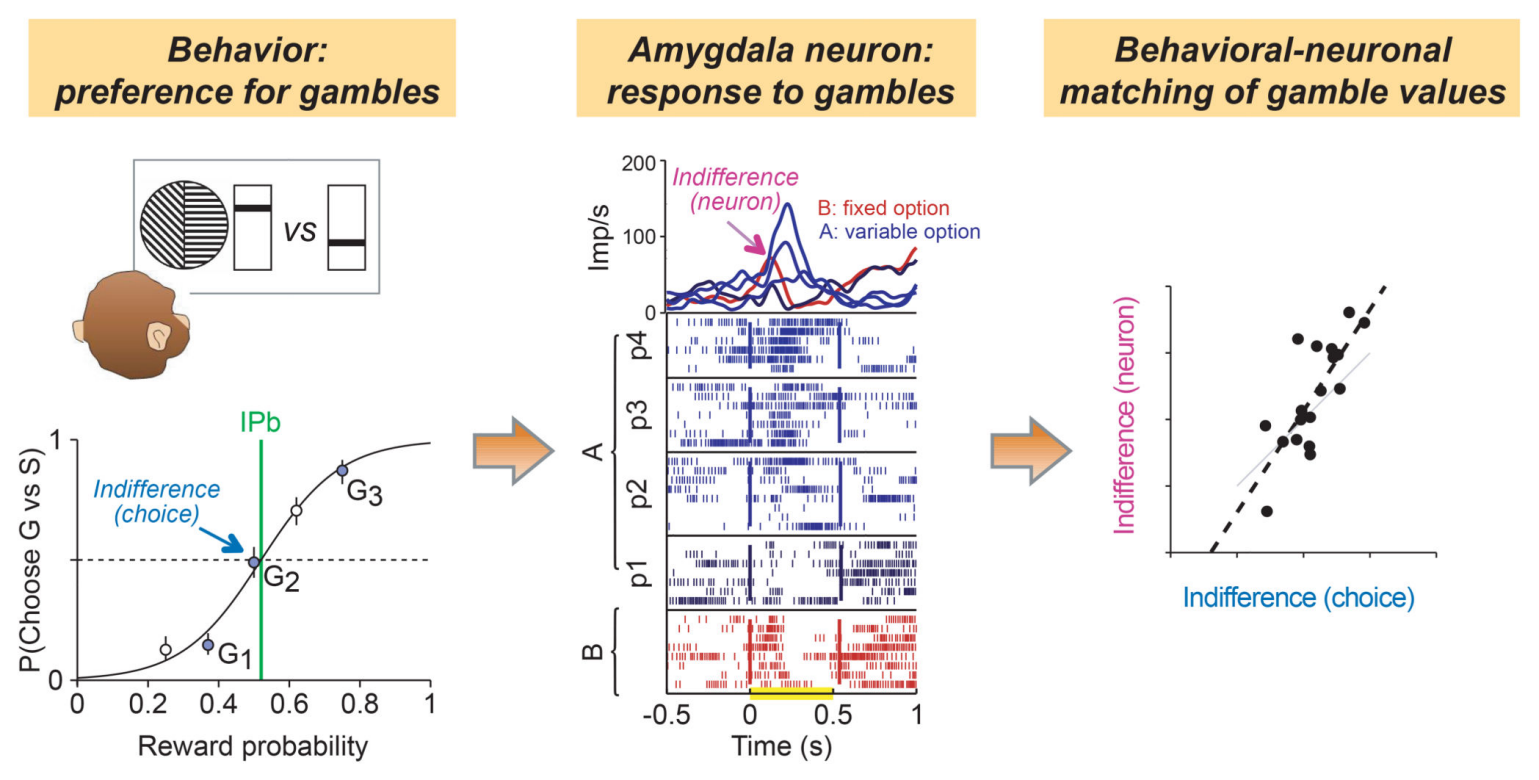


## New & Noteworthy

The amygdala—long associated with emotional responses—has recently been implicated in economic decision-making. Here we show that primate amygdala neurons respond to visual cues indicating reward probability and magnitude in a manner that reflects the integration of these variables into subjective values. These value signals complied with the notion that decision makers maximize the value of rewards, formalized by key axioms of economic theory. Beyond valuation, some neurons processed subjective values into choice-predictive signals.
