# Supplementary material for: Value coding by primate amygdala neurons complies with the continuity axiom of economic choice theory
Source: J Neurophysiol. Author manuscript; Available in PMC 2026 Mar 7. (PMC7618829; doi:10.1152/jn.00574.2025)
Supplement: Supplementary Material [file EMS212558-supplement-Supplementary_Material.pdf]

SUPPLEMENTARY FIGURES

Supplementary figures can be accessed under https://doi.org/10.6084/m9.figshare.31129393
